# Comparison of Immunogenicity and Protective Efficacy of PPR Live Attenuated Vaccines (Nigeria 75/1 and Sungri 96) Administered by Intranasal and Subcutaneous Routes

**DOI:** 10.3390/vaccines8020168

**Published:** 2020-04-06

**Authors:** Mana Mahapatra, M. Selvaraj, Satya Parida

**Affiliations:** The Pirbright Institute, Ash Road, Woking, Surrey GU24 0NF, UK; mana.mahapatra@pirbright.ac.uk (M.M.); muni.narayananselvaraj@pirbright.ac.uk (M.S.)

**Keywords:** peste des petits ruminants, vaccines, subcutaneous vaccination, intranasal vaccination, immune responses, real-time RT-PCR, virus neutralizing antibody titer

## Abstract

Following the successful eradication of rinderpest, the World Organization of Animal Health (OIE) and the Food and Agriculture Organization (FAO) have set a goal to eradicate peste des petits ruminants (PPR) globally by 2030. Vaccination is being taken forward as the key strategy along with epidemiological surveillance to target vaccination efforts and eradicate the disease. PPR is highly contagious and is generally spread by aerosolized droplets and close contact. Currently, two live attenuated vaccines (Nigeria 75/1 and Sungri 96) are in use, and administered subcutaneously to prevent transmission of PPR and protect vaccinated animals. Though the target cells that support primary replication of PPR vaccine strains are largely unknown, it is hypothesized that the immune response could be intensified following intranasal vaccine delivery as this route mimics the natural route of infection. This study aims to compare the immunogenicity and protective efficacy of the two currently available live attenuated PPR vaccines following subcutaneous and intranasal routes of vaccination in target species. Groups of five goats were vaccinated with live attenuated PPR vaccines (Nigeria 75/1 and Sungri 96) by either the subcutaneous or intranasal route, and 28 days later challenged intranasally with virulent PPR virus. All vaccinated animals regardless of vaccination route produced PPRV-specific antibodies post-vaccination. Following challenge, all goats were protected from clinical disease, and vaccination was considered to have induced sterilizing immunity. This study demonstrates that the intranasal route of vaccination is as effective as the subcutaneous route of vaccination when using available live attenuated PPR vaccines.

## 1. Introduction

Peste des petits ruminants (PPR) is a severe viral disease of small ruminants and many wild *Capra* species caused by the small ruminant morbillivirus, PPR virus (PPRV), closely related to rinderpest virus, in the family *Paramyxoviridae*. Since its first identification in the early 1940s in west Africa in Côte d’Ivoire, PPR has steadily expanded its geographical distribution, and currently large parts of sub-Saharan Africa, the whole of North Africa, the Middle East, and Asia except South–East Asia are endemic for PPR [1,2,3]. It is estimated that 330 million farmers across Africa, the Middle East, and Asia keep livestock [4], and that small ruminants play an important role in the livelihoods and food security of poor families, providing financial income and food security through provision of milk, meat, and various animal products. The annual impact of PPR alone is predicted to cost between 1.45 and 2.1 billion USD per year, of which about a third is borne by Africa, with a further quarter borne by South Asia [4]. This burden could be lessened with the successful eradication of PPR. Indeed, following the successful eradication of the closely related rinderpest virus, the World Organization for Animal Health (OIE) and the Food and Agriculture Organization (FAO) have targeted the global eradication of PPR by 2030. The initial phase of any eradication program requires an efficacious vaccine accompanied by robust diagnostic tests. Two live attenuated PPR vaccines are currently available; Nigeria 75/1 (lineage II origin) and Sungri 96 (lineage IV origin). These vaccines have been routinely employed subcutaneously in sheep and goats in endemic countries with great success [3,5], and have recently been shown to be capable of protecting goats against infection with field strains of all four genetic lineages [6]. 

Like other morbilliviruses, PPRV is both lympho- and epitheliotropic in nature and is mainly spread by aerosol droplets and, as such, often requires close contact between animals to spread within a herd [1,7]. It is believed that PPRV, like other morbilliviruses, initially infects epithelial cells of the respiratory tract. However, studies have suggested that immune cells present in the respiratory mucosa may take up PPR virions from the lumen of the respiratory tract and migrate to the T cell-rich areas of local lymphoid organs for the preliminary replication [8]. Although the PPR vaccines provide long-term immunity, a minimum of three years, the generation of virus neutralizing antibodies is not rapid enough to prevent the dissemination of the disease among in-contact animals [9]. Though the target cells that support replication of live attenuated PPRV are largely unknown, it is hypothesized that the immune response could be much faster following intranasal (i/n) vaccine delivery that mimics natural infection as reported in the case of other respiratory pathogens [10,11]. In addition, i/n vaccine delivery has the added advantage of being a non-invasive and environmental friendly method, and as such is easier to administer in the field, an obvious advantage during mass vaccination campaigns. Indeed, strong defensive cellular responses in the respiratory tract have been observed following i/n PPR vaccination [12] although to the best of our knowledge no systematic study has been undertaken to establish if i/n vaccination is equivalent or superior to the standard subcutaneous (s/c) route in providing sterile immunity. Therefore, the main aim of this study was to carry out a direct comparison of the immunogenicity and protective efficacy of PPR vaccines (Nigeria 75/1 and Sungri 96) following i/n or s/c vaccine delivery in target species.

## 2. Materials and Methods

### 2.1. Cells and Viruses 

Vero cells or VeroDogSLAM tag cells constitutively expressing the canine morbillivirus receptor SLAM (VDS) were used to grow PPRV, and undertake virus titration and virus neutralization tests. The cells were maintained in Dulbecco’s modified Eagle’s medium (DMEM) supplemented with 25 mM HEPES (pH 7.2) with 10 % (v/v) fetal calf serum (FCS, Gibco, Waltham, MA, USA), penicillin (100 Units/mL, Sigma, St. Louis, MO, USA), and streptomycin (100 μg/mL, Sigma, St. Louis, MO, USA). 

Two live attenuated PPR vaccine viruses, PPRV/Nigeria 75/1 and PPRV/Sungri 96 (written as Nigeria 75/1 and Sungri 96, respectively throughout this paper), were used to vaccinate the animals in this study. The challenge viruses used were PPRV/Morocco/2008 and PPRV/Ghana/78 as described previously [13]. Cells at about 60%–70% confluency were infected with the vaccine viruses at an multiplicity of infection (MOI) of 0.1 and incubated at 37 °C with 5% CO_2_. When virus induced cytopathic effect (CPE) was evident across the cell monolayer, virus was harvested by one cycle of freezing and thawing followed by centrifugation and stored at −70 °C as single-use aliquots.

### 2.2. Ethics Statement

Animal experiments were conducted under project license 70/6907 issued by the UK Home Office in accordance with relevant legislation and after approval by the Pirbright Institute (TPI) Animal Welfare and Ethical Review Board (AWERB), Pirbright, UK. 

### 2.3. Animal Studies

Two animal experiments were carried out in high containment facilities at the Pirbright Institute, United Kingdom, where animals were vaccinated with either the vaccine strain Nigeria 75/1 (lineage II) or Sungri 96 (lineage IV) and challenged with a wild-type PPRV (Table 1). For each animal experiment, fifteen mixed breeds of apparently healthy British goats of either sex, aged 6–9 months, were sourced from known commercial farms. Animals in each study were kept for at least a week prior to the start of the experiment for acclimatization to the new husbandry regime. During that time, all the animals were found to be healthy and free of disease, with average rectal temperatures ranging from 38.5–39.5 °C. For each vaccine under assessment, animals were randomly divided into three groups, with five animals in each group. Groups I and II received a 2 mL dose of vaccine (2 × 10^5^ tissue culture infectious doses (TCID_50_)) by s/c or i/n route, respectively. A further group (group III) served as the mock vaccinated control to ensure adequate challenge. The vaccine in the i/n group (II) was administered (1 ml to each nostril) using the LMA^®^ MAD Nasal™ Intranasal Mucosal Atomization Device (LMA^®^ MAD Nasal™) (LMA, San Diego, CA, USA) as described previously [14]. The LMA^®^ MAD Nasal™ device used for i/n vaccination or challenge comes as a soft conical plug that forms a seal with the nostril preventing expulsion of the vaccine. The spray atomizes the vaccine virus into a fine mist of particles that is delivered to the nasal mucosa. After four weeks, all the animals were challenged intranasally with 2 × 10^5^ TCID_50_ virulent PPRV (lineage II PPRV/Ghana/78 for challenging lineage IV Sungri 96 vaccinated goats, and lineage IV PPRV/Morocco/2008 for challenging lineage II Nigeria 75/1 vaccinated goats) to assess the clinical protection afforded by the existing vaccines following heterologous challenge. Following challenge, the animals were observed at least twice daily with clinical signs and rectal temperatures being recorded for up to 14 days. Animals in the control group were treated with antibiotics to avoid secondary infection, and the animals which developed severe clinical signs were humanely euthanized according to an established clinical scorecard [8]. Blood (ethylenediamine tetraacetic acid (EDTA)/heparinized and clotted blood) samples were collected on various days post-vaccination (dpv) and challenge (dpc) (Table 1). Heparinized blood samples were used for leucocyte counts and peripheral blood mononuclear cell (PBMC) separation, whereas EDTA blood was used for the detection of PPRV nucleic acid by real-time RT-PCR assay. Serum was separated from clotted blood and stored at −20 °C until use. Ocular, nasal, and saliva swabs were collected weekly following vaccination and at regular time points following challenge, and were stored at −70 °C until processed (Table 1).

### 2.4. Assays for PPRV Anti-N Antibodies

The development of antibodies against PPRV N-protein was measured using a commercially available ELISA kit (IDVet, Grabels, France) following the manufacturer’s instruction. In this assay, percent inhibition values less than 50 are considered positive. All the samples were run in duplicate, and the samples showing positive in only one well were repeat-tested for confirmation. 

### 2.5. Virus Neutralizing Antibody Titer

The development of PPRV neutralizing antibodies was measured using a virus neutralization test (VNT) as previously described [15]. The 50% neutralizing end points were calculated using the method as described by Reed and Muench [16] and expressed as log_10_ VN_50_/mL. A neutralizing titer of greater than 10 is considered positive. The tests were performed at least twice and the average of the two tests was used for subsequent analysis.

### 2.6. RNA Extraction and Detection of PPRV Nucleic Acid in Blood and Swabs Samples

Total RNA was extracted from the clinical material (nasal, mouth, and eye swabs, and EDTA-blood) as described previously [13] and stored at −70 °C as single-use aliquots until tested. All samples were analyzed by real-time RT-PCR (RT-qPCR) to detect the presence of PPRV nucleic acid [17] using the Superscript III Platinum R one step qRT-PCR system kit (Invitrogen, Carlsbad, CA, USA)) on the ABI 7500 system (Applied Biosystems, Paisely, UK). All samples were run in duplicate, and the samples showing positive in only one well were repeat-tested for confirmation.

### 2.7. Statistical Analysis

Data were analyzed using mixed models in which the vaccine used and time were fixed factors, and individual animals were random factors. Two-way ANOVA followed by Sidak multiple comparison tests was performed using GraphPad Prism version 8.0.1 for Windows (GraphPad Software, La Jolla California, CA, USA, www.graphpad.com; accessed on 19 December 2019). Temperature profiles, leucocyte counts, and 40-C_T_ values were analyzed separately for each vaccine experiment. Temperature profiles and leucocyte counts were fit to the general linear model; p-values less than 0.05 were considered statistically significant.

## 3. Results

### 3.1. Clinical Observations

**Figure 1:**
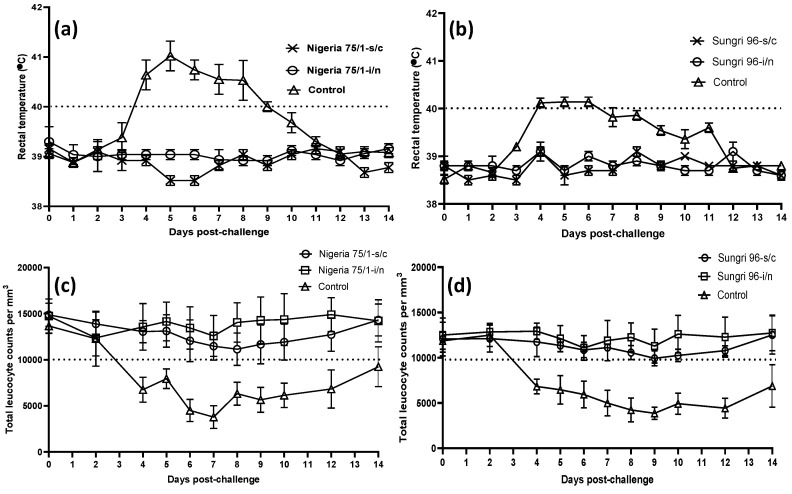
Average rectal temperature and leucocyte count in goats following challenge with virulent peste des petits ruminants virus (PPRV). Average rectal temperature and leucocyte count of five animals in each group are shown in the graph. (**a**) Average rectal temperature in Nigeria 75/1 vaccine study; (**b**) average rectal temperature in Sungri 96 vaccine study; (**c**): average leucocyte count in Nigeria 75/1 vaccine study; (**d**) average leucocyte count in Sungri 96 vaccine study. The dotted line in each graph shows the normal body temperature/leucocyte count.

During the acclimatization period (first week following arrival) animals had rectal temperatures within the normal range, 38.5 to 39.5 °C (data not shown). Following vaccination, none of the animals showed any adverse reaction. The temperature of all of the vaccinated animals before challenge remained within the normal range (data not shown), and in the majority of vaccinated animals, there was an initial leukocytosis on day 2 followed by a slight decline in leukocyte count up to day 8 (data not shown), but the count always remained within the normal range for goats. Following i/n challenge, the animals in the vaccinated groups did not show any rise in body temperature or leucopenia (Figure 1). There were no PPRV-specific clinical signs in vaccinated animals except for a slight watery nasal discharge in one animal in the Nigeria 75/1 i/n group and two animals in the Sungri 96 i/n group on 10 dpc, and lacrimation in two animals in Sungri 96 s/c group on 7–8 dpc. In contrast, all the control animals were clinically infected with a rise in body temperature that started on 3 dpc, reaching more than 40 °C for the majority of animals by 4 dpc (Figure 1a). The infected animals in the Morocco/2008 challenge group reached a peak pyrexia of >41 °C on 5 dpc and remained pyrexic with temperatures exceeding 40 °C until 8 dpc (Figure 1a). Animals infected with the Ghana/78 virus exhibited a peak pyrexia of >40 °C on 5 dpc and remained pyrexic up to 6 dpc, after which temperatures started to decline, although two animals (G-7742 and G-7872) remained pyrexic until 8 dpc (Figure 1b). Nasal discharge was observed in all mock vaccinated infected animals at 3–4 dpc that became muco-purulent by 5–8 dpc in most cases. There was congestion of nasal and buccal mucosae, ocular discharge, and appearance of mouth/dental pad lesions from day 4–6, which persisted for a further 7 days, prior to convalescence. Some animals demonstrated inappetance, indicating oral trauma, and mouth lesions were observed. Animals in the control groups developed a pronounced lecuopenia post-challenge, with leucocyte counts reaching as low as ~4000 leucocytes/mm^3^ of blood by 6–7 dpc for lineage IV (Morocco/2008 strain) and 9 dpc for lineage II PPRV (Ghana/78 strain), with a gradual recovery in leucocyte numbers observed after this point for those not terminated according to the clinical score sheet (data not shown). One animal (G-4231) from the Morocco/2008 challenge group and three animals (G-380, G-7742, G-7812) from the Ghana/78 challenge group were humanely killed on 7 and 10 dpc, respectively, having reached humane endpoints as defined in the clinical score system.

### 3.2. Detection of Viral Nucleic Acids in Blood and Swabs

**Figure 2:**
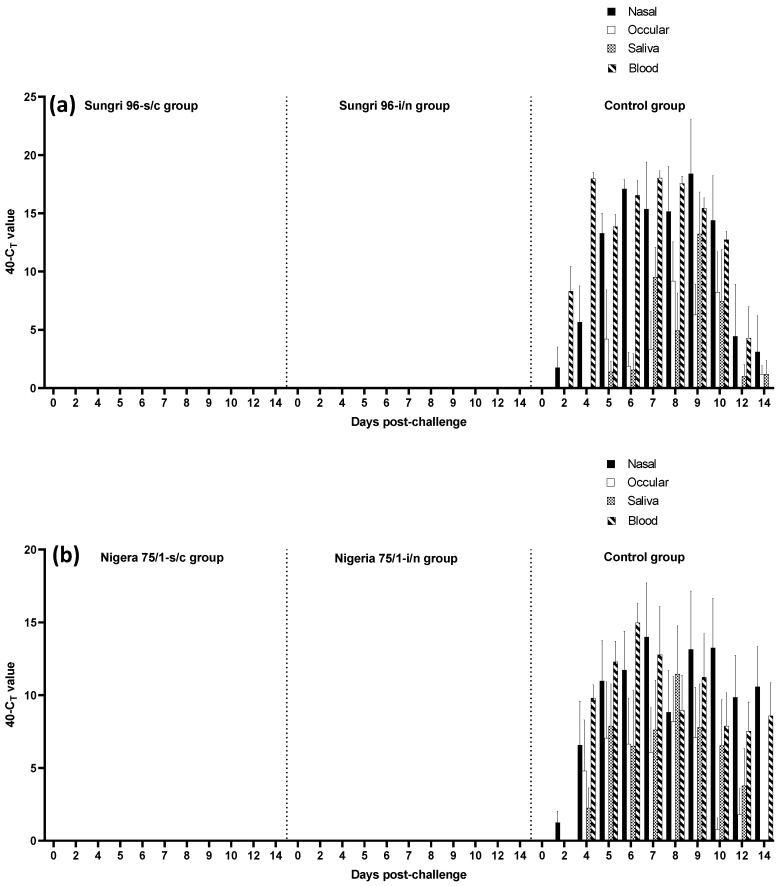
PPRV-specific RNA was measured by reverse-transcription—real-time PCR (RT-qPCR) and the amount of viral RNA is expressed as the mean 40-*C_T_* value, a value which increases as the amount of viral RNA increases. Average values for five animals in each group on different days post-challenge are shown. (**a**) Nigeria 75/1 vaccine study; (**b**) Sungri 96 vaccine study.

Blood and swab samples were collected from the animals following vaccination and challenge, and RT-qPCR was carried out to detect viral nucleic acids in blood and various body excretions. No viral nucleic acid was detected in any of the vaccinated animals following vaccination or challenge indicating that the vaccines were safe to use and provided sterile immunity by both routes of vaccination (Figure 2a,b). In contrast, viral nucleic acid was detected in all sample types taken from unvaccinated control animals in both the studies. The Ghana/78 unvaccinated challenge group had viral nucleic acid being detected in the nasal swabs and blood from 2 dpc onwards whereas viral nucleic acid was detected in ocular and saliva swabs on 5 dpc onwards until the end of the experiment. Similarly, in the case of the Morocco/2008 challenge group, viral nucleic acid was detected in nasal swabs from 2 dpc onwards whereas viral nucleic acid started appearing in ocular and saliva swabs and blood a little bit later, i.e., 4 dpc onwards (Figure 2a,b).

### 3.3. Antibody Response against the Viral Nucleocapsid Protein

**Figure 3:**
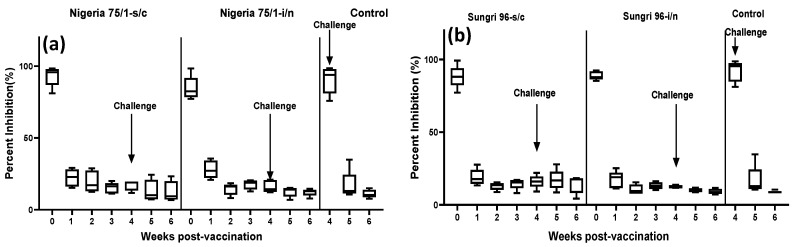
Anti-N antibody response following vaccination and challenge as measured by N-protein based cELISA. The results are presented as percent inhibition values. The data are presented as box-and-whisker plots, in which the bars span the minimum and maximum values of five animals, and the box shows the range from the first to the third quartile. The central horizontal line in each box shows the median value. Day of challenge is shown by an arrow in each graph. (**a**): Percent inhibition values in Nigeria 75/1 vaccine study; (**b**) percent inhibition values in Sungri 96 vaccine study.

Following vaccination, antibodies directed to the viral nucleocapsid (N) protein were measured using a commercially available ELISA kit. On the day of vaccination (day 0), all animals were sero-negative for anti-N antibodies (Figure 3a,b). Both vaccines delivered either by the s/c or i/n route, induced antibodies that reacted strongly in the N-protein based cELISA. By one-week post-vaccination, animals in both s/c and i/n group in both vaccine studies had equivalent levels of detectable anti-N antibody that increased by two weeks post-vaccination and then was maintained throughout the remaining period of the study (Figure 3a,b). On the day of challenge (4 weeks post-vaccination), the animals in the i/n group in Sungri 96 and Nigeria 75/1 vaccine studies exhibited slightly higher levels of anti-N antibody compared to the respective s/c group. Statistical analysis by two way ANOVA was carried out to compare the level of anti-N antibody between the s/c and i/n group as a whole for all the collection time points, however no statistical significance was observed (p = 0.5034 for Nigeria 75/1 and p = 0.129 for Sungri 96). Further statistical analysis to compare the weekly antibody responses of the s/c group with that of the i/n group of Nigeria 75/1 vaccinated animals revealed no statistical significance (p = 0.5825, p = 0.7455, p = 0.8322, p = 0.9368, p = 0.9999, p = 0.9999. and p = 0.9999 for 0, 1, 2, 3, 4, 5, and 6 week post-vaccination, respectively) in any group at any time point. Similarly, for Sungri 96 vaccinates when the weekly anti-N antibody response of the s/c group was compared with that of the i/n group no statistical significance was observed (p = 0.9999, p = 0.9995, p = 0.9117, p = 0.994, p = 0.7154, p = 0.4859, and p = 0.6751 for 0, 1, 2, 3, 4, 5, and 6 week post-vaccination, respectively) in any group at any time point. Following challenge, no anamnestic response was observed in any vaccinated animals. 

### 3.4. Neutralizing Antibody Titres

**Figure 4:**
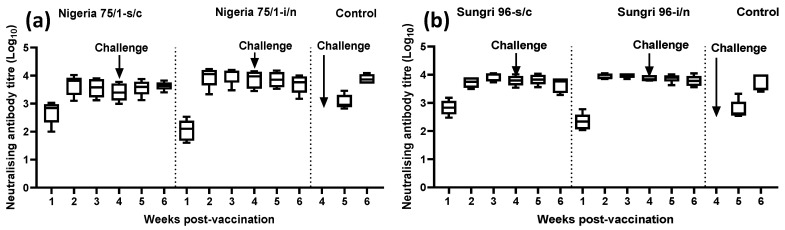
Neutralizing antibody response in goats following vaccination and challenge. The data are presented as box-and-whisker plots, in which the bars span the minimum and maximum values of five animals, and the box shows the range from the first to the third quartile. The central horizontal line in each box shows the median value. Day of challenge is shown by an arrow in each graph. (**a**) Neutralizing antibody titers in Nigeria 75/1 vaccine study; (**b**): neutralizing antibody titers in Sungri 96 vaccine study.

Virus neutralization assays were carried out to determine the virus-neutralizing antibody titers in the serum of vaccinated and control animals before and after challenge with virulent PPRV. In this study, homologous virus was used for the neutralization assay, i.e., Nigeria 75/1 virus for the Nigeria 75/1 vaccine study and Sungri 96 virus for the Sungri 96 vaccine study. As expected for British goats on day 0, all of the animals were negative for the presence of PPRV-specific neutralizing antibody. In both the vaccine studies, there was detectable level of neutralizing antibody in vaccinated animals by one week post-vaccination which increased by two weeks post-vaccination and then plateaued for the remaining period of the study (Figure 4a,b). The neutralizing antibody level in the i/n group was slightly lower than the s/c group on one week post-vaccination; however was found to be slightly higher on 2-, 3-, 4-, 5-, and 6-weeks post-vaccination. No neutralizing antibody was detected in two animals in the Sungri 96 i/n group on one-week post-vaccination; similarly two animals in the Nigeria 75/1 i/n group had low neutralizing antibody (log_10_ 1.75 and 1.85) on one-week post-vaccination. During vaccine administration the animals in the i/n group sneezed/shook their heads which may have resulted in loss of some vaccine. However, when the level of mean neutralizing antibody was compared between the s/c and i/n group by two-way ANOVA, no statistically significant difference was observed (p = 0.3972 for Nigeria 75/1 and p = 0.9897 for Sungri 96). Similarly, when the weekly neutralizing antibody response of the s/c group with that of the i/n group was compared for Nigeria 75/1 vaccinated animals, no statistical significance was observed (p = 0.1765 for 1st week; p = 0.7813 for 2nd week; p = 0.3029 for 3rd week; p = 0.282 for 4th week; p = 0.5744 for 5th week; p = 0.9991 for 6th week) in any group at any time point. Similarly, when the weekly neutralizing antibody responses of the s/c group with that of the i/n group were compared for Sungri 96 vaccinated animals, no statistical significance was observed (p = 0.1169 for 1st week; p = 0.3474 for 2nd week; p = 0.9973 for 3rd week; p = 0.9467 for 4th week; p = 0.9991 for 5th week and p = 0.9345 for 6th week) in any group at any time point. No neutralizing antibody was detected in any of the control animals on the day of challenge; however, they exhibited equivalent levels of neutralizing antibody by two weeks post-challenge (Figure 4a,b). 

## 4. Discussion

PPR remains a significant threat to the establishment of sustainable agriculture and food security in areas where the virus is endemic. Vaccination is the key to preventing and controlling PPR in high risk or endemic areas. The chances of PPR eradication success are directly linked to the ability to vaccinate the vast majority of small ruminants, and this can be a significant challenge in smallholder village production systems because of the low density of small ruminants or in very remote areas where vaccine supply can be problematic. The quality and adaptability of vaccine delivery systems is also a key element that impacts on strategic vaccine implementation [4]. One of the key factors for the success of the global rinderpest eradication program was the use of a rinderpest (TCRV Plowright) vaccine that was highly efficacious in protecting animals against all rinderpest strains [18]. A similar tool also exists for the prevention and control of PPR. Indeed, two efficient live attenuated PPR vaccines (Nigeria 75/1 and Sungri 96) are available that are usually administered subcutaneously, and can induce lifelong protective immunity [3,5,19]. Nigeria 75/1 (lineage II) vaccine is used all over endemic regions whereas Sungri 96 (lineage IV) vaccine is mainly used across India. There is evidence for the successful use of Nigeria 75/1 vaccine in controlling lineage IV field outbreaks in China in 2007 and 2013 [20] and Morocco in 2008 [21]. In a previous study, goats vaccinated via s/c route with lineage II Nigeria 75/1 vaccine were protected from s/c or i/n challenge with virulent Ivory Coast strain (lineage I) [14,22]. In this study, groups of goats were vaccinated either subcutaneously or intranasally with Nigeria 75/1 and Sungri 96 vaccines, and subsequently challenged intranasally with lineage IV virulent virus (Morocco/2008) and lineage II virulent virus (Ghana/78), respectively, with all animals being protected without any excretion of virus in body secretions. Therefore, it is clear that these two vaccines used either subcutaneously or intranasally can provide sterile immunity against an i/n heterologous challenge. Recently, Hodgson and colleagues used s/c route as the route of vaccination and challenge, and reported these two vaccines to be effective at providing sterile immunity against infection from all four lineages of PPRV [6]. Therefore, it is clear that both the existing PPR vaccines could be used to control the disease globally irrespective of circulation of any lineage of the virus.

Currently, these live attenuated vaccines are thermolabile, and require maintenance of the cold chain until administration [19], although work on thermostable PPR vaccine is on-going [23]. The commercially available vaccines are currently obtained in freeze-dried form usually as 100 doses per vial, and are reported to be stable for at least two years at 2 °C to 8 °C and for several years at –20 °C. However, following reconstitution the vaccine needs to be administered quickly, preferably within 30 minutes [4]. PPR is mainly endemic in tropical countries across Asia, the Middle East, and Africa, and some of these countries have a poor veterinary infrastructure where maintenance of the cold chain in the field, a factor critical to preserve vaccine quality to obtain expected potency and efficacy, is problematic. Therefore, a quick and easy administration route for vaccination via the non-invasive i/n route may be helpful, and preferred during the mass vaccination campaigns. Intranasal PPR vaccination has been reported to generate strong mucosal and defensive cellular responses in the respiratory tract compared to vaccination via the s/c and intramuscular (i/m) route [12]. In addition, it is expected that where the natural route of infection is adopted for vaccination it may enhance the immune response through vaccine virus triggering the same immune signaling pathway as a natural infection. Certainly this approach has been observed for measles and influenza vaccination [10,11]. Flumist is a licensed, intranasally-administered live attenuated influenza virus vaccine that has been shown to stimulate both mucosal and systemic immunity. An analysis of the results of the clinical trials conducted in children comparing the efficacy of Flumist with inactivated vaccine or placebo suggests that the intranasally administered vaccine was more effective in preventing influenza [11]. Therefore, we hypothesized at the beginning of the work that i/n vaccination might elicit immune response relatively quickly compared to the conventional route (s/c) of vaccination.

In the current study, we immunized two groups of goats for each vaccine, one by the s/c route and the second by the i/n route and studied their immunogenicity and protective efficacy following vaccination and challenge. A LMA^®^ MAD Nasal™ device was used for i/n vaccination or challenge that atomizes the vaccine/challenge virus into a fine mist of particles of 30 to 100 microns in size, delivered directly to the nasal mucosa, mimicking natural infection. Following vaccination, no PPRV nucleic acid was detected in any body excretions in any vaccinated animals. Similar observations were reported by Enchery and colleagues and Hodgson and colleagues where the vaccine was administered by s/c route [6,24]. As expected, all the vaccinated animals in both the groups were protected from i/n challenge with virulent PPRV indicating both routes of vaccination to be equally efficient. Further, following i/n challenge no PPRV nucleic acid was detected in any of the body excretions of the vaccinated animals in our studies indicating the vaccines provided sterile immunity. Aerosol measles vaccination in non-human primates [25,26] and aerosol MMR vaccination in children [27,28] were reported to be comparable to those vaccinated by injection. 

In a recent study, Hodgson and colleagues did not observe anti-N antibody responses or neutralizing antibody response at one-week post-vaccination [6] whereas in our study we observed detectable amounts of anti-N antibody and also neutralizing antibody in both the s/c and i/n groups. This variation could be the effect of one log less vaccine dose administered to the animals in the previous study (2 × 10^4^ TCID_50_ versus 2 × 10^5^ TCID_50_ in the current study). Similarly, Enchery and colleagues could detect anti-N antibodies only at 9 dpv [24] where the goats were vaccinated with PPR-VAC, a live vaccine based on the Nigeria 75/1 attenuated strain.

The lower neutralizing antibody level in two goats of i/n group compared to the s/c group on the 1st week post-vaccination could be ascribed to the sneezing of the animals in the i/n group during the administration of the vaccine. However, all the animals vaccinated by the i/n route showed higher or at least equivalent neutralizing-antibody titers compared to the s/c group on 2nd, 3rd, and 4th week of vaccination, and the difference was not found to be statistically significant. In a measles virus study, aerosol measles vaccination has been reported to be equivalent or superior to s/c injection [10]. Measles antibody levels and the proportion of seropositive children after six-years post-vaccination were found to be significantly higher in the aerosol group compared to the group that received vaccine subcutaneously. They recommended measles re-vaccination by the aerosol route as it evokes a stronger and much longer lasting antibody response than the injected vaccine [10]. 

In conclusion, the hypothesis that i/n vaccination of animals with PPR vaccine can induce early immune response was not reflected in neutralizing antibody response, although compared to the s/c group a higher level of neutralizing antibody was observed on 2, 3, and 4 weeks post-vaccination. It is to be noted that the i/n vaccination has the added difficulty of the animals shaking their heads/sneezing following vaccine administration that may result in insufficient dosage of the vaccine. However, i/n vaccination has the added value of being a non-invasive method of vaccination, and environmental friendly because of the needle-free delivery of the vaccine. As there is no statistically significant difference in the immune responses between the two routes of vaccination for both the vaccines in goats, it is advisable to continue the s/c administration of PPR vaccines during mass vaccination campaigns until long-term vaccination studies are carried out in sheep and goats to further evaluate i/n route of vaccination. Certainly, long-term studies involving i/n PPRV vaccine in the target species are warranted.

## Figures and Tables

**Table 1:** Design of the animal experiment. s/c: subcutaneous; i/n: intranasal; dpc: days post-challenge.

Vaccine Study	Group	Vaccination Route	Challenge (i/n)	Samples Collected	Days of Sample Collection
Nigeria 75/1	Gr-I	s/c	Morocco/2008	Blood, Swabs (nasal, ocular, and saliva)	0, 1, 2, 3, and 4 weeks post-vaccination; 2, 4, 5, 6, 7, 8, 9, 10, 12, 14 dpc
	Gr-II	i/n			
	Gr-III	Control			
Sungri 96	Gr-I	s/c	Ghana/78	Blood, Swabs (nasal, ocular, and saliva)	0, 1, 2, 3, and 4 weeks post-vaccination; 2, 4, 5, 6, 7, 8, 9, 10, 12, 14 dpc
	Gr-II	i/n			
	Gr-III	Control			
